# Open and closed valve commissural fusion after biventricular assist device implantation

**DOI:** 10.1093/ehjcr/ytaa406

**Published:** 2020-11-26

**Authors:** Kazuhiro Kamada, Toru Hashimoto, Akira Shiose, Hiroyuki Tsutsui

**Affiliations:** 1 Department of Cardiovascular Medicine, Faculty of Medical Sciences, Kyushu University, 3-1-1 Maidashi, Higashi-ku, Fukuoka, Fukuoka 812-8582, Japan; 2 Department of Advanced Cardiopulmonary Failure, Faculty of Medical Sciences, Kyushu University, 3-1-1 Maidashi, Higashi-ku, Fukuoka, Fukuoka 812-8582, Japan; 3 Department of Cardiovascular Surgery, Faculty of Medical Sciences, Kyushu University, 3-1-1 Maidashi, Higashi-ku, Fukuoka, Fukuoka 812-8582, Japan

A 29-year-old woman with idiopathic dilated cardiomyopathy was implanted with durable left ventricular assist device (LVAD, Jarvik 2000) as a bridge-to-transplantation. She repeatedly experienced heart failure hospitalization due to aortic regurgitation (AR) and right ventricular (RV) dysfunction since 2 years post-LVAD. The heart failure symptoms got refractory three and half years post-LVAD. Echocardiography demonstrated severe AR (Supplementary material online, *Figure S1*), akinesis of the RV, and incomplete closure of the tricuspid and pulmonary valves due to marked RV dilatation (Supplementary material online, *Figure S2*). She underwent conversion to paracorporeal continuous-flow biventricular assist device (monopivot centrifugal pumps) with replacement of both aortic and pulmonary valves to prevent valve insufficiency (INSPIRIS bioprosthetic valves, 19 and 21 mm for each); the pulmonary valve replacement was necessitated because of incomplete valve closure. However, she re-developed symptomatic heart failure—systemic congestion with kidney and liver dysfunction—in 2 months. Echocardiography revealed severe pulmonary regurgitation (PR) (Supplementary material online, *Figure S3*). Since aggressive diuresis and extracorporeal ultrafiltration were ineffective, the circuit system configuration was converted to central extracorporeal membrane oxygenation to resolve recirculation within the RV assist device (RVAD); drainage from the right atrium, pulmonary artery, and left ventricle, and return to the aorta (Supplementary material online, *Figure S4*). Although the heart failure was finally controlled, she soon died of cerebrovascular accident. Autopsy revealed contrasting valve commissural fusion; the fixed-closed aortic valve (*Figure 1*) and the fixed-open pulmonary valve to which PR was attributable (*Figure 2*). Aortic regurgitation is among causes of post-LVAD heart failure; the contributing factors are inadequate LV unloading, permanently closed AV, commissural fusion, changes in valve leaflets, systemic hypertension, and support period.^1^^,^^2^ Post-RVAD PR has not been described except one report, in which PR in the native pulmonary valve caused only haemolysis but not heart failure.^3^ Despite the prophylactic pulmonary valve replacement, severe PR rapidly developed due to pulmonary valve commissural fusion at the fixed-open position in the present case. The mechanisms of post-RVAD PR are unproved, but anastomotic position and angle between the outflow graft and the pulmonary trunk, requirement of high RVAD flow due to RV akinesis, and diminished pulsatility might contribute to the progressive PR.

**Figure 1:**
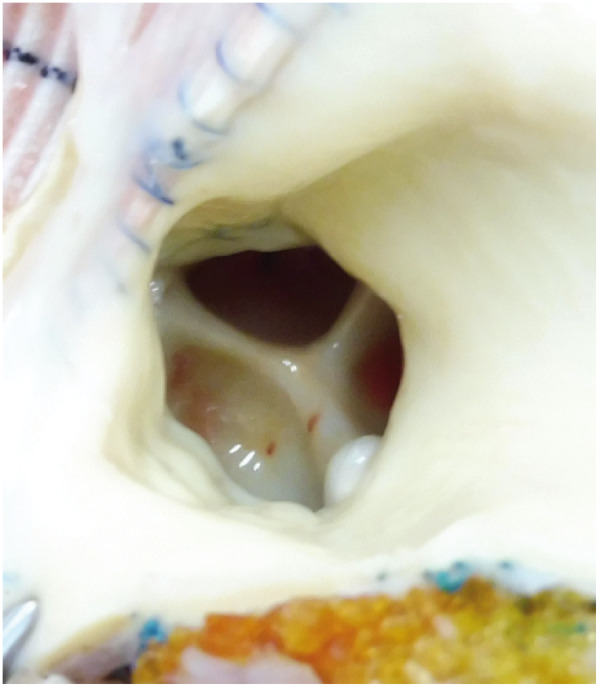
Autopsy findings of the aortic bioprosthetic valve. The aortic valve was fixed-closed due to commissural fusion.

**Figure 2:**
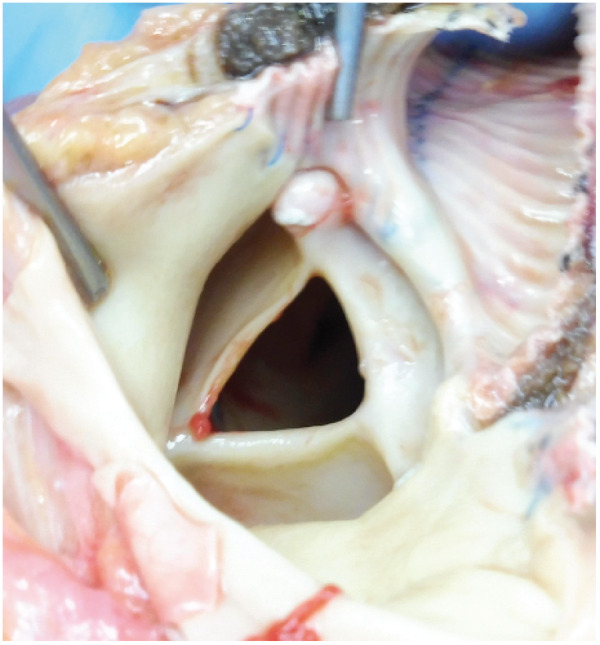
Autopsy findings of the pulmonary bioprosthetic valve. The pulmonary valve was fixed-open due to commissural fusion.

## Supplementary material

Supplementary material is available at *European Heart Journal - Case Reports* online.

**Consent:** The Informed consent for submission and publication was obtained from the parents of the patient because she was deceased.

**Funding:** This study was supported by the JSPS KAKENHI grant 19K17567 to T.H.

**Conflict of interest:** none declared.

## Supplementary Material

ytaa406_Supplementary_Data
