# Supplementary material for: Open and closed valve commissural fusion after biventricular assist device implantation
Source: Eur Heart J Case Rep. 2020 Nov 26;4(6):1–2. doi: 10.1093/ehjcr/ytaa406 (PMC7793159; doi:10.1093/ehjcr/ytaa406)

**Supplementary Figure and Figure Legend**

**Supplementary Figure 1. Transthoracic echocardiography of aortic regurgitation after LVAD implantation.** The parasternal long-axis views with or without color flow Doppler were shown. Doppler echocardiography demonstrated severe aortic regurgitation (arrowheads). AoV indicates aortic valve; LV, left ventricle; RV, right ventricle.

**
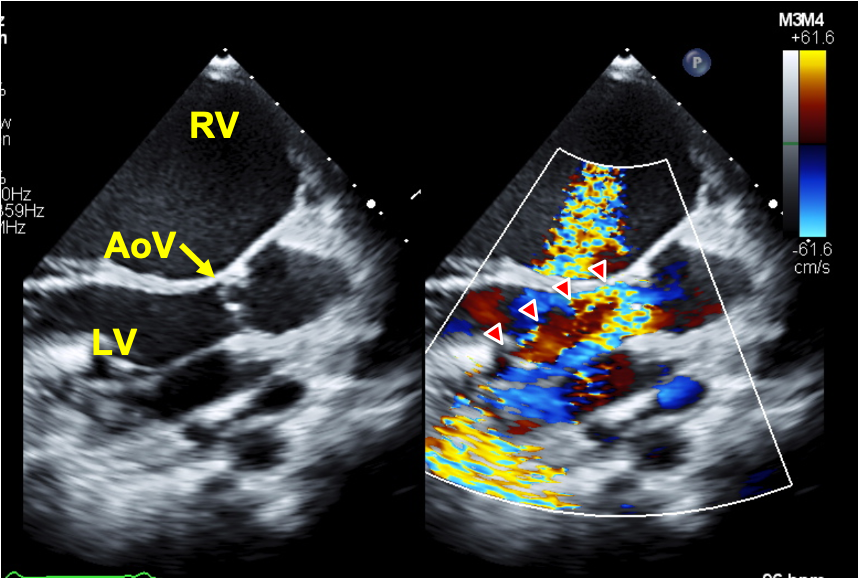
**

**Supplementary Figure 2. Transthoracic echocardiography of pre-LVAD and after development of post-LVAD heart failure.** Apical 4-chamber view (A) and parasternal short axis view showing aortic and pulmonary valves (B) before LVAD implantation. Marked RV enlargement overwhelming LV (C), and incomplete closure of the pulmonary valve due to severe RV dilatation (D) were observed three years post-LVAD implantation. AoV indicates aortic valve; PV, pulmonary valve; LV, left ventricle; RV, right ventricle; LA left atrium; RA, right atrium; PA, pulmonary artery.

**
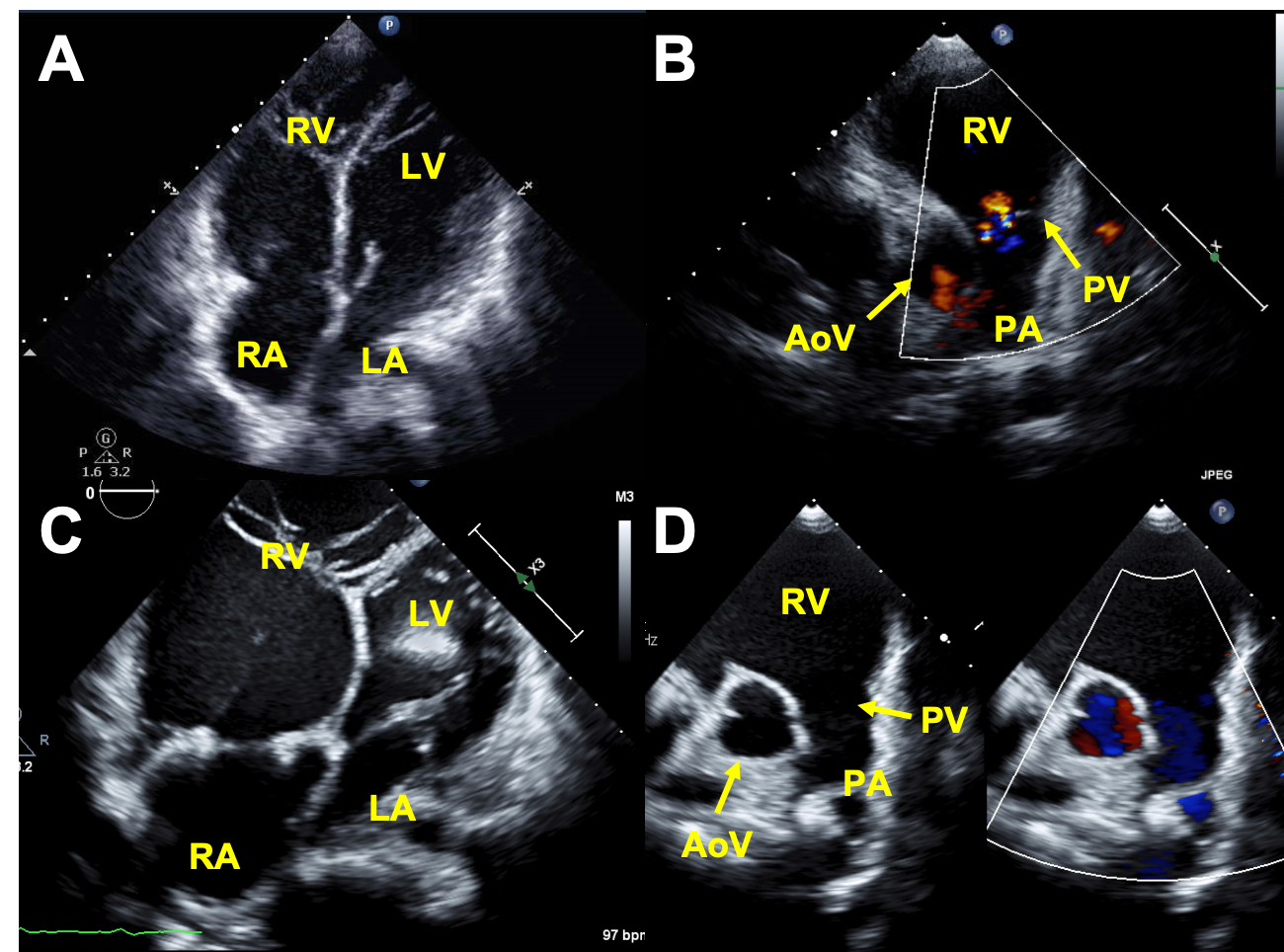
**

**Supplementary Figure 3. Transthoracic echocardiographic images.** The parasternal short-axis views with or without color flow Doppler were shown. Doppler echocardiography demonstrated severe pulmonary regurgitation (arrowheads). AoV indicates aortic valve; PV, pulmonary valve; PA, pulmonary artery; RV, right ventricle.


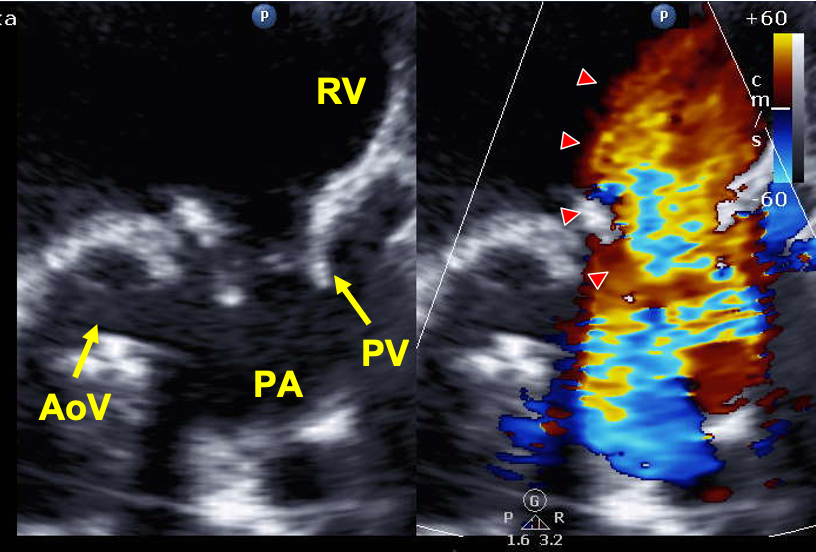


**Supplementary Figure 3. Illustration of mechanical circulatory support devices used in the present case.** Durable LVAD was removed and paracorporeal biventricular assist device (BiVAD) was implanted because of severe aortic regurgitation and right ventricular dysfunction. After relapse of right heart failure due to severe pulmonary regurgitation, the circuit system configuration was converted to central extracorporeal membrane oxygenation (ECMO) to diminish recirculation within the right ventricular assist device (RVAD); drainage from the right atrium, pulmonary artery, and left ventricle, and return to the aorta.


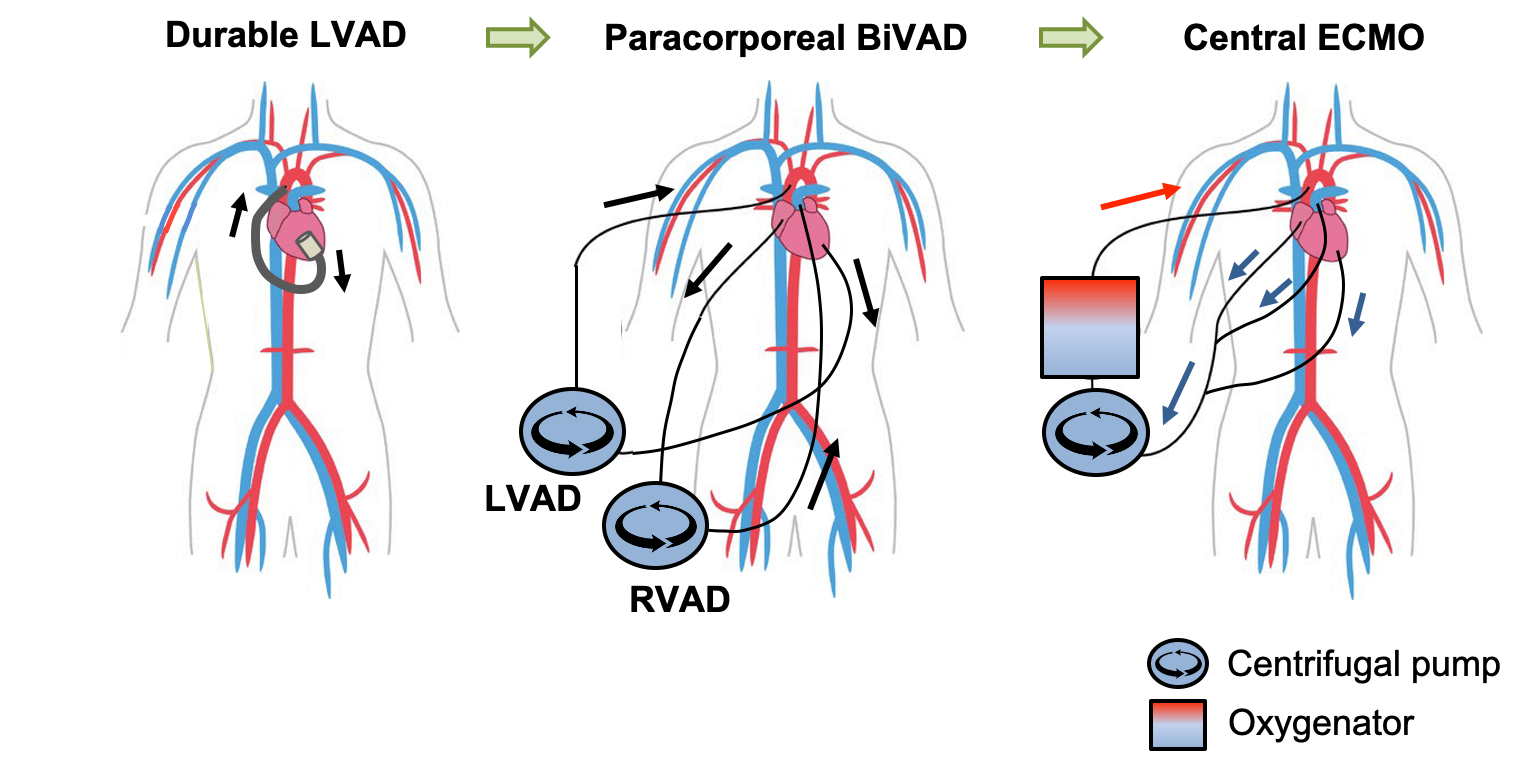

Supplement: ytaa406_Supplementary_Data [file ytaa406_supplementary_data.docx]
